# Isotypic crystal structures of 1-benzyl-4-(4-bromo­phen­yl)-2-imino-1,2,5,6,7,8,9,10-octa­hydro­cyclo­octa­[*b*]pyridine-3-carbo­nitrile and 1-benzyl-4-(4-fluoro­phen­yl)-2-imino-1,2,5,6,7,8,9,10-octa­hydro­cyclo­octa­[*b*]pyridine-3-carbo­nitrile

**DOI:** 10.1107/S1600536814022016

**Published:** 2014-10-18

**Authors:** R. A. Nagalakshmi, J. Suresh, S. Maharani, R. Ranjith Kumar, P. L. Nilantha Lakshman

**Affiliations:** a Department of Physics, The Madura College, Madurai 625 011, India; b Department of Organic Chemistry, School of Chemistry, Madurai Kamaraj University, Madurai 625 021, India; c Department of Food Science and Technology, University of Ruhuna, Mapalana, Kamburupitiya 81100, Sri Lanka

**Keywords:** crystal structure, cyclo­octa­pyridine, hydrogen bonding

## Abstract

The mol­ecules of the two isotypic title compounds, C_25_H_24_BrN_3_, (I), and C_25_H_24_FN_3_, (II), comprise a 2-imino­pyridine ring fused with a cyclo­octane ring. In (I), the cyclo­octane ring adopts a twisted chair–chair conformation, while in (II), this ring adopts a twisted boat–chair conformation. The dihedral angles between the planes of the pyridine ring and the bromo­benzene and phenyl rings are 80.14 (12) and 71.72 (13)°, respectively, in (I). The equivalent angles in (II) are 75.25 (8) and 68.34 (9)°, respectively. In both crystals, inversion dimers linked by pairs of C—H⋯N inter­actions generate *R*_2_^2^(14) loops, which are further connected by weak C—H⋯π inter­actions, generating (110) sheets.

## Abstract 2

Two isotypic title compounds comprise a 2-imino­pyridine ring fused with a cyclo­octane ring. In one compound, the cyclo­octane ring adopts a twisted chair–chair conformation, while in the second, this ring adopts a twisted boat–chair conformation.

## Chemical context   

The pyridine skeleton is of great importance to chemists as well as to biologists as it is found in a large variety of naturally occurring compounds and also in clinically useful mol­ecules having diverse biological activities. Its derivatives are known to possess anti­microbial (Jo *et al.*, 2004 ▶) and anti­viral (Mavel *et al.*, 2002 ▶) activities. The heterocyclic 1,4-di­hydro­pyridine ring is a common feature in compounds with various pharmacological activities such as anti­microbial (Hooper *et al.*, 1982 ▶) and anti­thrombotic (Sunkel *et al.*, 1990 ▶) activities. The chemistry of imines in particular is of special inter­est in the literature due to their numerous practical applications (Echevarria *et al.*, 1999 ▶). Imines have attracted much attention because of their wide variety of applications in the electronics and photonics fields (Wang *et al.*, 2001 ▶). Imines and their complexes have a variety of applications in the biological, clinical and analytical fields (Singh *et al.*, 1975 ▶; Patel *et al.*, 1999 ▶). Our inter­est in the preparation of pharmacologically active 2-imino pyridines led us to synthesise the title compounds and we have undertaken the X-ray crystal structure determination of these compounds in order to establish their conformations.

## Structural commentary   

**Figure 1:**
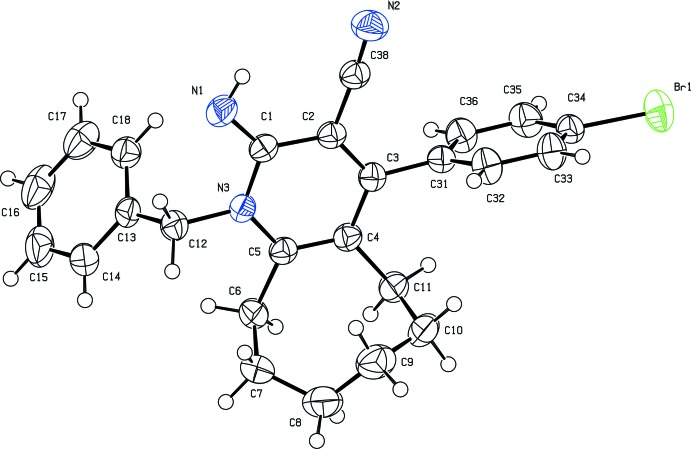
The mol­ecular structure of (I), showing 50% probability displacement ellipsoids.

The structures of compounds (I) and (II) are shown in Figs. 1 ▶ and 2, respectively ▶. The cyclo­octane ring adopts a twisted chair–chair conformation in compound (I) and twisted boat–chair conformation (Wiberg, 2003 ▶) in compound (II).
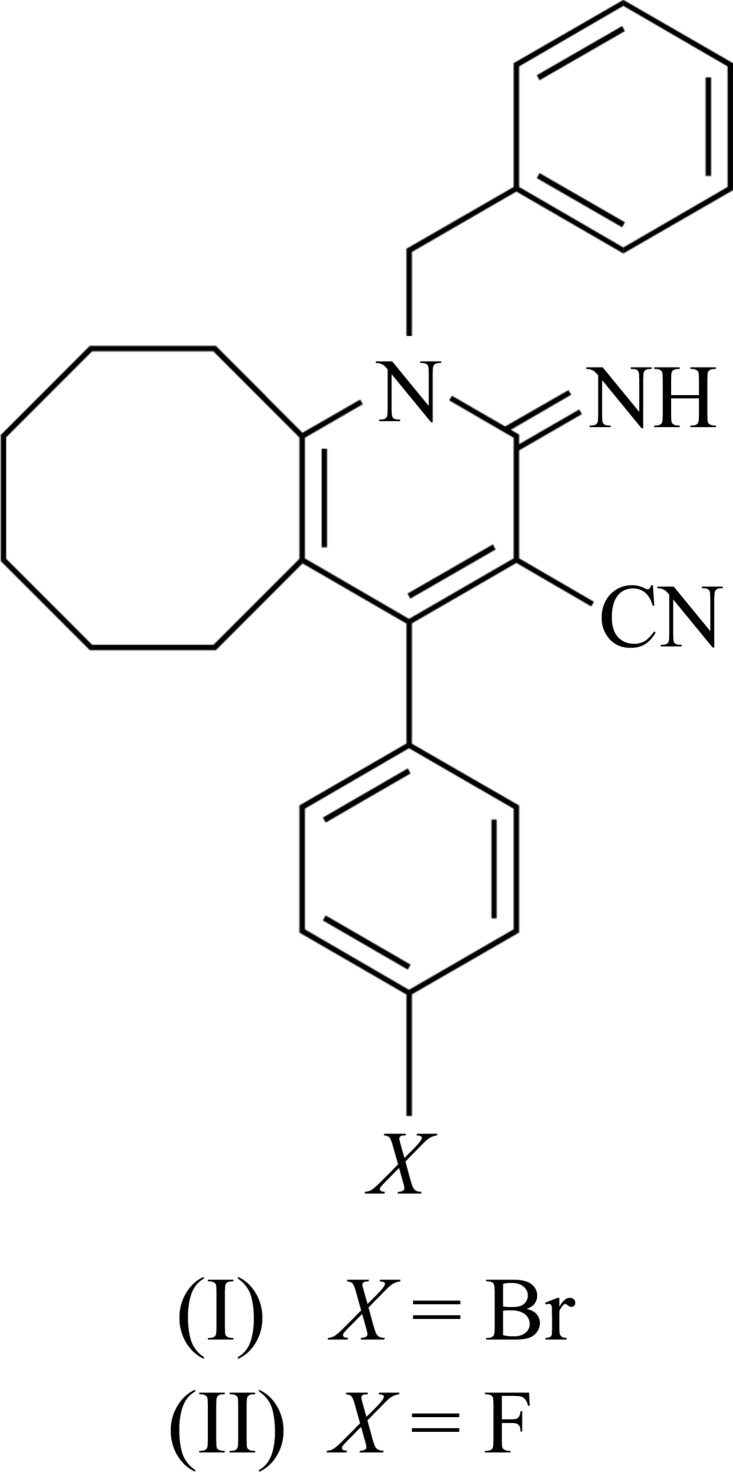


 In both compounds, the imino group is nearly coplanar with the pyridine ring, as indicated by the N1=C1—N3—C5 torsion angle [178.8 (2) for compound (I) and 179.05 (13)° for compound (II)]. Steric hindrances rotate the phenyl (C13–C18) and aromatic (C31–C36) rings out of the plane of the central pyridine ring by 71.72 (13) and 80.14 (12)°, respectively, in compound (I), and by 68.34 (9) and 75.25 (8)°, respectively, in compound (II). Opening up of the N3—C5—C4 angle [121.54 (19)° for compound (I) and 121.29 (13)° for compound (II)] and considerable shortening of the C5—N3 [1.376 (3) Å for compound (I) and 1.3777 (18) Å for compound (II)] bond distance may directly be attributed to the bulky substituents at the *ortho* position C5. The endocyclic angles of the pyridine ring cover the range 114.29 (18)–123.02 (2)° and 118.86 (13)–123.11 (12)° for compounds (I) and (II) respectively. The C1—N3—C5 angle [122.93 (2) for compound (I) and 123.11 (12)° for compound (II)] is expanded as in pyridine itself [123.9 (3)°; Jin *et al.*, 2005 ▶].

## Supramol­ecular features   

**Figure 3:**
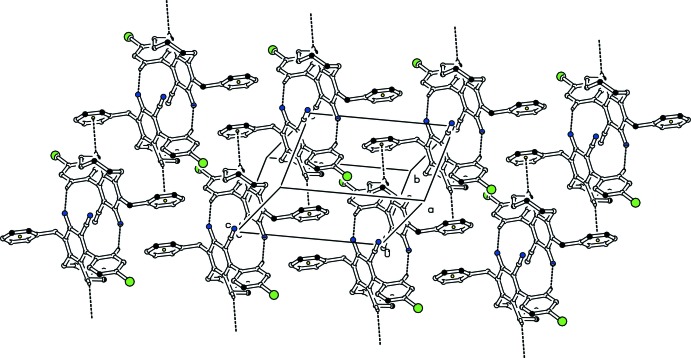
Partial packing diagram of the title compound (I). Dashed lines represent inter­molecular hydrogen bonds and C—H⋯π contacts. For clarity, H atoms not involved in hydrogen bonding have been omitted.

In the crystals, pairs of C—H⋯N inter­actions form 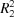
(14) ring motifs (Bernstein *et al.*, 1995 ▶), and the resulting dimers are further connected through weak C—H⋯π inter­actions involving the phenyl ring as acceptor (Tables 1 ▶ and 2 ▶, Figs. 3 ▶, 4 ▶). In each case, the resulting supra­molecular structure is a layer propagating parallel to the (110) plane.

## Database survey   

Similar structures reported in the literature are 2-meth­oxy-4-(2-meth­oxy­phen­yl)-5,6,7,8,9,10-hexa­hydro­cyclo­octa­[*b*]pyr­idine-3-carbo­nitrile (Vishnupriya *et al.*, 2014*a* ▶) and 4-(2-fluorophen­yl)-2-meth­oxy-5,6,7,8,9,10-hexa­hydro­cyclo­octa­[*b*]-pyridine-3-carbo­nitrile (Vishnupriya *et al.*, 2014*b* ▶). The twisted conformation of the cyclo­octane ring of compound (I) is similar to those found in the related structures. However, the C=NH functional group present in the title compound allows the formation of C—H⋯N hydrogen bonds, which are not present in the above-cited compounds. In the title compounds, the bond lengths in the central pyridine ring span the range 1.369–1.446 Å, which compare well with the range observed in the similar structures (1.314–1.400 Å), but these bonds are systematically longer in the title compounds, due to the substitution of the pyridine N atom by a benzyl group. The bond length of the nitrile group attached to pyridine ring [N2  C38 = 1.137 (3) Å in compound (I) and 1.1426 (19) Å in compound (II)] and the linearity of the cyano moiety [N2 C38—C2 = 176.3 (3) for compound (I) and 175.68 (17)° for compound (II)] have characteristic features that are observed in 3-cyano-2-pyridine derivatives (Hursthouse *et al.*, 1992 ▶; Patel *et al.*, 2002 ▶).

## Synthesis and crystallization   

The two compounds were prepared in a similar manner using 4-fluoro aldehyde (1 mmol) for compound (I) and 4-bromo aldehyde (1 mmol) for compound (II). A mixture of cyclo­octa­none (1mmol), respective aldehyde (1 mmol) and malono­nitrile (1 mmol) were taken in ethanol (10 mL) to which *p*-toluene­sulfonic acid (pTSA) (0.5 mmol) was added. The reaction mixture was heated under reflux for 2–3 h. After completion of the reaction (TLC), the reaction mixture was poured into crushed ice and extracted with ethyl acetate. The excess solvent was removed under vacuum and the residue was subjected to column chromatography using petroleum ether/ethyl acetate mixture (97:3 *v*/*v*) as eluent to afford pure product. The product was recrystallized from ethyl acetate, affording colourless crystals of compounds (I) and (II) [m.p. 493 K; yield 91% for (I) and m.p. 473 K; yield 65% for (II)].

## Refinement   

Crystal data, data collection and structure refinement details are summarized in Table 3 ▶. C-bound H atoms were placed in calculated positions and allowed to ride on their carrier atoms, with C—H = 0.93 (aromatic CH) or 0.97 Å (methyl­ene CH_2_). Imine atom H1 was found in a difference map and refined with a distance restraint in both compounds of N—H = 0.86 (10) Å. Isotropic displacement parameters for H atoms were calculated as *U*_iso_ = 1.5*U*_eq_(C) for CH_3_ groups and *U*_iso_ = 1.2*U*_eq_(carrier atom) for all other H atoms. The DELU restraint was applied in compound (II).

## Supplementary Material

Crystal structure: contains datablock(s) global, I, II. DOI: 10.1107/S1600536814022016/hb7284sup1.cif

Structure factors: contains datablock(s) I. DOI: 10.1107/S1600536814022016/hb7284Isup2.hkl

Structure factors: contains datablock(s) II. DOI: 10.1107/S1600536814022016/hb7284IIsup3.hkl

Supporting information file. DOI: 10.1107/S1600536814022016/hb7284Isup4.cml

Supporting information file. DOI: 10.1107/S1600536814022016/hb7284IIsup5.cml

CCDC references: 1027782, 1027783

Additional supporting information:  crystallographic information; 3D view; checkCIF report

## Figures and Tables

**Figure 2:**
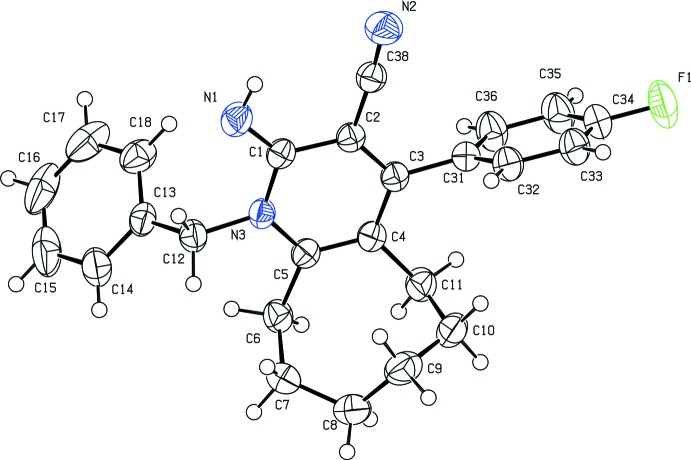
The mol­ecular structure of (II), showing 50% probability displacement ellipsoids.

**Figure 4:**
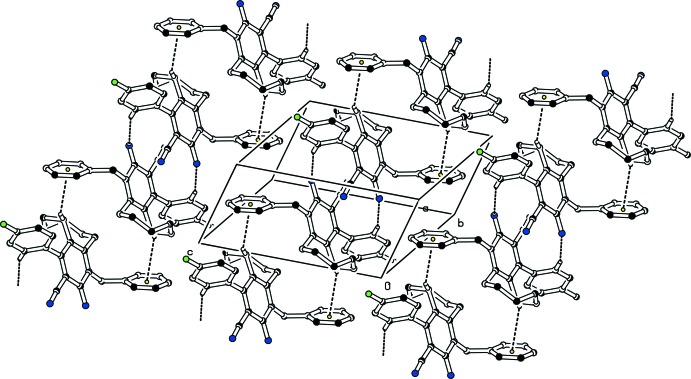
Partial packing diagram of the title compound (II). Dashed lines represent inter­molecular hydrogen bonds and C—H⋯π contacts. For clarity, H atoms not involved in hydrogen bonding have been omitted.

**Table 1:** Hydrogen-bond geometry (, ) for (I) *Cg*1 is the centroid of the C13C18 phenyl ring.

*D*H*A*	*D*H	H*A*	*D* *A*	*D*H*A*
C32H32N1^i^	0.93	2.56	3.421(3)	154
C11H11*A* *Cg*1^ii^	0.97	2.97	3.648(3)	128

Symmetry codes: (i) 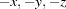; (ii) 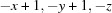.

**Table 2:** Hydrogen-bond geometry (, ) for (II) *Cg*1 is the centroid of the C13C18 phenyl ring.

*D*H*A*	*D*H	H*A*	*D* *A*	*D*H*A*
C32H32N1^i^	0.93	2.53	3.421(2)	160
C11H11*A* *Cg*1^ii^	0.97	2.93	3.484(2)	118

Symmetry codes: (i) 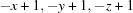; (ii) 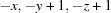.

**Table 3:** Experimental details

	(I)	(II)
Crystal data		
Chemical formula	C_25_H_24_BrN_3_	C_25_H_24_FN_3_
*M*_r_	446.38	385.47
Crystal system, space group	Triclinic, *P* 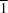	Triclinic, *P* 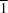
Temperature (K)	293	293
*a*, *b*, *c* ()	10.2103(3), 10.7643(4), 11.6942(4)	10.1370(4), 10.2078(3), 11.8238(4)
, , ()	101.074(1), 106.726(1), 115.058(1)	109.688(2), 100.309(2), 111.420(2)
*V* (^3^)	1039.46(6)	1006.73(6)
*Z*	2	2
Radiation type	Mo *K*	Mo *K*
(mm^1^)	1.99	0.08
Crystal size (mm)	0.21 0.19 0.18	0.21 0.19 0.18

Data collection		
Diffractometer	Bruker Kappa APEXII	Bruker Kappa APEXII
Absorption correction	Multi-scan (*SADABS*; Bruker, 2004 ▶)	Multi-scan (*SADABS*; Bruker, 2004 ▶)
*T*_min_, *T*_max_	0.967, 0.974	0.967, 0.974
No. of measured, independent and observed [*I* > 2(*I*)] reflections	25106, 4532, 3830	23254, 3752, 2876
*R*_int_	0.027	0.022
(sin /)_max_ (^1^)	0.639	0.606

Refinement		
*R*[*F* ^2^ > 2(*F* ^2^)], *wR*(*F* ^2^), *S*	0.039, 0.107, 1.03	0.039, 0.109, 1.05
No. of reflections	4532	3752
No. of parameters	266	267
No. of restraints	2	2
H-atom treatment	H atoms treated by a mixture of independent and constrained refinement	H atoms treated by a mixture of independent and constrained refinement
_max_, _min_ (e ^3^)	0.93, 0.87	0.17, 0.14

Computer programs: *APEX2* and *SAINT* (Bruker, 2004 ▶), *SHELXS97*, *SHELXL97* and *SHELXL2014*/6 (Sheldrick, 2008 ▶) and *PLATON* (Spek, 2009 ▶).

## supplementary crystallographic information

### Crystal data

**Table:**

C_25_H_24_FN_3_	*Z* = 2
*M**_r_* = 385.47	*F*(000) = 408
Triclinic, *P*1	*D*_x_ = 1.272 Mg m^−^^3^
*a* = 10.1370 (4) Å	Mo *K*α radiation, λ = 0.71073 Å
*b* = 10.2078 (3) Å	Cell parameters from 2000 reflections
*c* = 11.8238 (4) Å	θ = 2–31°
α = 109.688 (2)°	µ = 0.08 mm^−^^1^
β = 100.309 (2)°	*T* = 293 K
γ = 111.420 (2)°	Block, colourless
*V* = 1006.73 (6) Å^3^	0.21 × 0.19 × 0.18 mm

### Data collection

**Table:**

Bruker Kappa APEXII diffractometer	2876 reflections with *I* > 2σ(*I*)
Radiation source: fine-focus sealed tube	*R*_int_ = 0.022
φ and ω scans	θ_max_ = 25.5°, θ_min_ = 2.3°
Absorption correction: multi-scan (*SADABS*; Bruker, 2004)	*h* = −12→12
*T*_min_ = 0.967, *T*_max_ = 0.974	*k* = −12→12
23254 measured reflections	*l* = −14→14
3752 independent reflections	

### Refinement

**Table:**

Refinement on *F*^2^	Hydrogen site location: mixed
Least-squares matrix: full	H atoms treated by a mixture of independent and constrained refinement
*R*[*F*^2^ > 2σ(*F*^2^)] = 0.039	*w* = 1/[σ^2^(*F*_o_^2^) + (0.0436*P*)^2^ + 0.3339*P*] where *P* = (*F*_o_^2^ + 2*F*_c_^2^)/3
*wR*(*F*^2^) = 0.109	(Δ/σ)_max_ < 0.001
*S* = 1.05	Δρ_max_ = 0.17 e Å^−^^3^
3752 reflections	Δρ_min_ = −0.14 e Å^−^^3^
267 parameters	Extinction correction: *SHELXL2014*/6 (Sheldrick, 2008), Fc^*^=kFc[1+0.001xFc^2^λ^3^/sin(2θ)]^-1/4^
2 restraints	Extinction coefficient: 0.027 (3)

### Special details

**Table:**

Geometry. All e.s.d.'s (except the e.s.d. in the dihedral angle between two l.s. planes) are estimated using the full covariance matrix. The cell e.s.d.'s are taken into account individually in the estimation of e.s.d.'s in distances, angles and torsion angles; correlations between e.s.d.'s in cell parameters are only used when they are defined by crystal symmetry. An approximate (isotropic) treatment of cell e.s.d.'s is used for estimating e.s.d.'s involving l.s. planes.

### Fractional atomic coordinates and isotropic or equivalent isotropic displacement parameters (Å^2^)

**Table:**

	*x*	*y*	*z*	*U*_iso_*/*U*_eq_	
C1	0.38640 (16)	0.41969 (17)	0.59999 (13)	0.0347 (3)	
C2	0.28977 (16)	0.44165 (16)	0.50992 (13)	0.0343 (3)	
C3	0.16525 (16)	0.32042 (16)	0.40896 (13)	0.0341 (3)	
C4	0.12688 (16)	0.16454 (17)	0.39051 (13)	0.0366 (3)	
C5	0.21646 (16)	0.13987 (16)	0.47452 (13)	0.0345 (3)	
C6	0.18252 (19)	−0.02211 (18)	0.46013 (15)	0.0442 (4)	
H6A	0.0738	−0.0860	0.4268	0.053*	
H6B	0.2196	−0.0159	0.5442	0.053*	
C7	0.2506 (2)	−0.1046 (2)	0.37256 (17)	0.0562 (5)	
H7A	0.3563	−0.0323	0.3975	0.067*	
H7B	0.2469	−0.1927	0.3887	0.067*	
C8	0.1783 (2)	−0.1646 (2)	0.22930 (17)	0.0583 (5)	
H8A	0.2219	−0.2277	0.1857	0.070*	
H8B	0.0716	−0.2328	0.2046	0.070*	
C9	0.1946 (2)	−0.0406 (2)	0.18178 (17)	0.0570 (5)	
H9A	0.2813	0.0570	0.2441	0.068*	
H9B	0.2155	−0.0717	0.1026	0.068*	
C10	0.0585 (2)	−0.0109 (2)	0.15827 (15)	0.0564 (5)	
H10A	−0.0238	−0.1036	0.0864	0.068*	
H10B	0.0830	0.0743	0.1337	0.068*	
C11	0.00304 (18)	0.02948 (18)	0.27088 (15)	0.0475 (4)	
H11A	−0.0777	0.0557	0.2488	0.057*	
H11B	−0.0377	−0.0616	0.2872	0.057*	
C12	0.44774 (17)	0.23479 (19)	0.65601 (14)	0.0411 (4)	
H12A	0.5478	0.3219	0.6876	0.049*	
H12B	0.4517	0.1414	0.6027	0.049*	
C13	0.41019 (17)	0.21501 (18)	0.76831 (14)	0.0409 (4)	
C14	0.4226 (2)	0.0975 (2)	0.79530 (17)	0.0578 (5)	
H14	0.4469	0.0274	0.7406	0.069*	
C15	0.3991 (2)	0.0836 (3)	0.9035 (2)	0.0756 (7)	
H15	0.4071	0.0040	0.9208	0.091*	
C16	0.3641 (3)	0.1865 (3)	0.98465 (19)	0.0782 (7)	
H16	0.3500	0.1780	1.0580	0.094*	
C17	0.3499 (2)	0.3013 (2)	0.95823 (17)	0.0694 (6)	
H17	0.3251	0.3706	1.0133	0.083*	
C18	0.3719 (2)	0.31600 (19)	0.85034 (16)	0.0521 (4)	
H18	0.3609	0.3944	0.8328	0.063*	
C31	0.07281 (16)	0.35321 (17)	0.31963 (13)	0.0371 (3)	
C32	0.12480 (18)	0.39428 (18)	0.22997 (15)	0.0435 (4)	
H32	0.2186	0.4033	0.2270	0.052*	
C33	0.0391 (2)	0.4221 (2)	0.14484 (16)	0.0491 (4)	
H33	0.0734	0.4484	0.0839	0.059*	
C34	−0.09671 (19)	0.4101 (2)	0.15238 (16)	0.0494 (4)	
C35	−0.1521 (2)	0.3707 (2)	0.23943 (18)	0.0586 (5)	
H35	−0.2454	0.3637	0.2424	0.070*	
C36	−0.06628 (19)	0.3413 (2)	0.32320 (17)	0.0526 (4)	
H36	−0.1027	0.3133	0.3827	0.063*	
C38	0.33469 (17)	0.60054 (18)	0.53208 (14)	0.0395 (3)	
N1	0.50412 (15)	0.52710 (17)	0.69710 (13)	0.0485 (4)	
N2	0.37721 (18)	0.73047 (17)	0.55698 (14)	0.0572 (4)	
N3	0.34211 (13)	0.26298 (14)	0.57514 (11)	0.0346 (3)	
F1	−0.18123 (13)	0.43791 (15)	0.06937 (11)	0.0758 (4)	
H1	0.520 (2)	0.6177 (14)	0.7008 (19)	0.065 (6)*	

### Atomic displacement parameters (Å^2^)

**Table:**

	*U*^11^	*U*^22^	*U*^33^	*U*^12^	*U*^13^	*U*^23^
C1	0.0340 (7)	0.0397 (8)	0.0314 (7)	0.0162 (6)	0.0147 (6)	0.0157 (6)
C2	0.0359 (7)	0.0374 (7)	0.0328 (7)	0.0173 (6)	0.0154 (6)	0.0167 (6)
C3	0.0353 (7)	0.0403 (8)	0.0326 (7)	0.0194 (6)	0.0157 (6)	0.0181 (6)
C4	0.0347 (8)	0.0383 (8)	0.0350 (8)	0.0157 (6)	0.0112 (6)	0.0158 (6)
C5	0.0360 (7)	0.0376 (8)	0.0322 (7)	0.0162 (6)	0.0153 (6)	0.0167 (6)
C6	0.0515 (9)	0.0422 (8)	0.0411 (8)	0.0199 (7)	0.0139 (7)	0.0238 (7)
C7	0.0739 (12)	0.0487 (10)	0.0532 (10)	0.0376 (9)	0.0180 (9)	0.0221 (8)
C8	0.0773 (13)	0.0448 (9)	0.0506 (10)	0.0314 (9)	0.0204 (9)	0.0156 (8)
C9	0.0743 (12)	0.0491 (10)	0.0431 (9)	0.0251 (9)	0.0243 (9)	0.0169 (8)
C10	0.0729 (12)	0.0428 (9)	0.0354 (9)	0.0186 (9)	0.0039 (8)	0.0135 (7)
C11	0.0421 (9)	0.0407 (8)	0.0467 (9)	0.0137 (7)	0.0027 (7)	0.0172 (7)
C12	0.0405 (8)	0.0499 (9)	0.0373 (8)	0.0251 (7)	0.0112 (6)	0.0204 (7)
C13	0.0387 (8)	0.0425 (8)	0.0324 (7)	0.0133 (7)	0.0039 (6)	0.0164 (7)
C14	0.0618 (11)	0.0604 (11)	0.0516 (10)	0.0290 (9)	0.0076 (9)	0.0299 (9)
C15	0.0747 (14)	0.0776 (14)	0.0661 (13)	0.0202 (12)	0.0013 (11)	0.0504 (12)
C16	0.0799 (15)	0.0760 (15)	0.0375 (10)	−0.0012 (12)	0.0042 (10)	0.0284 (10)
C17	0.0788 (14)	0.0539 (11)	0.0429 (10)	0.0053 (10)	0.0240 (10)	0.0117 (9)
C18	0.0616 (11)	0.0412 (9)	0.0427 (9)	0.0140 (8)	0.0192 (8)	0.0164 (7)
C31	0.0391 (8)	0.0378 (8)	0.0346 (8)	0.0190 (6)	0.0110 (6)	0.0156 (6)
C32	0.0418 (8)	0.0477 (9)	0.0451 (9)	0.0207 (7)	0.0159 (7)	0.0246 (7)
C33	0.0565 (10)	0.0548 (10)	0.0469 (9)	0.0266 (8)	0.0200 (8)	0.0319 (8)
C34	0.0564 (10)	0.0532 (10)	0.0450 (9)	0.0318 (8)	0.0102 (8)	0.0250 (8)
C35	0.0529 (10)	0.0847 (13)	0.0617 (11)	0.0458 (10)	0.0251 (9)	0.0396 (10)
C36	0.0530 (10)	0.0756 (12)	0.0524 (10)	0.0389 (9)	0.0266 (8)	0.0389 (9)
C38	0.0427 (8)	0.0410 (8)	0.0368 (8)	0.0197 (7)	0.0155 (7)	0.0178 (7)
N1	0.0435 (8)	0.0441 (8)	0.0426 (8)	0.0135 (7)	0.0039 (6)	0.0149 (6)
N2	0.0693 (10)	0.0438 (8)	0.0571 (9)	0.0241 (7)	0.0215 (8)	0.0230 (7)
N3	0.0360 (6)	0.0407 (7)	0.0299 (6)	0.0189 (5)	0.0114 (5)	0.0168 (5)
F1	0.0799 (8)	0.1045 (9)	0.0715 (7)	0.0590 (7)	0.0180 (6)	0.0556 (7)

### Geometric parameters (Å, º)

**Table:**

C1—N1	1.2847 (19)	C12—N3	1.4790 (18)
C1—N3	1.3994 (18)	C12—C13	1.501 (2)
C1—C2	1.446 (2)	C12—H12A	0.9700
C2—C3	1.369 (2)	C12—H12B	0.9700
C2—C38	1.428 (2)	C13—C18	1.381 (2)
C3—C4	1.419 (2)	C13—C14	1.382 (2)
C3—C31	1.4896 (19)	C14—C15	1.385 (3)
C4—C5	1.3737 (19)	C14—H14	0.9300
C4—C11	1.505 (2)	C15—C16	1.366 (3)
C5—N3	1.3777 (18)	C15—H15	0.9300
C5—C6	1.502 (2)	C16—C17	1.358 (3)
C6—C7	1.533 (2)	C16—H16	0.9300
C6—H6A	0.9700	C17—C18	1.381 (2)
C6—H6B	0.9700	C17—H17	0.9300
C7—C8	1.520 (2)	C18—H18	0.9300
C7—H7A	0.9700	C31—C36	1.380 (2)
C7—H7B	0.9700	C31—C32	1.384 (2)
C8—C9	1.518 (2)	C32—C33	1.382 (2)
C8—H8A	0.9700	C32—H32	0.9300
C8—H8B	0.9700	C33—C34	1.358 (2)
C9—C10	1.517 (3)	C33—H33	0.9300
C9—H9A	0.9700	C34—F1	1.3570 (18)
C9—H9B	0.9700	C34—C35	1.363 (2)
C10—C11	1.525 (2)	C35—C36	1.382 (2)
C10—H10A	0.9700	C35—H35	0.9300
C10—H10B	0.9700	C36—H36	0.9300
C11—H11A	0.9700	C38—N2	1.1426 (19)
C11—H11B	0.9700	N1—H1	0.864 (9)
			
N1—C1—N3	118.86 (13)	C10—C11—H11B	109.2
N1—C1—C2	126.92 (14)	H11A—C11—H11B	107.9
N3—C1—C2	114.22 (12)	N3—C12—C13	115.71 (12)
C3—C2—C38	121.57 (13)	N3—C12—H12A	108.4
C3—C2—C1	123.31 (13)	C13—C12—H12A	108.4
C38—C2—C1	115.11 (13)	N3—C12—H12B	108.4
C2—C3—C4	119.20 (13)	C13—C12—H12B	108.4
C2—C3—C31	119.87 (13)	H12A—C12—H12B	107.4
C4—C3—C31	120.92 (13)	C18—C13—C14	118.55 (15)
C5—C4—C3	118.86 (13)	C18—C13—C12	122.21 (14)
C5—C4—C11	120.91 (13)	C14—C13—C12	119.14 (15)
C3—C4—C11	119.70 (13)	C13—C14—C15	120.31 (19)
C4—C5—N3	121.29 (13)	C13—C14—H14	119.8
C4—C5—C6	121.54 (13)	C15—C14—H14	119.8
N3—C5—C6	117.16 (12)	C16—C15—C14	120.2 (2)
C5—C6—C7	115.02 (13)	C16—C15—H15	119.9
C5—C6—H6A	108.5	C14—C15—H15	119.9
C7—C6—H6A	108.5	C17—C16—C15	119.92 (19)
C5—C6—H6B	108.5	C17—C16—H16	120.0
C7—C6—H6B	108.5	C15—C16—H16	120.0
H6A—C6—H6B	107.5	C16—C17—C18	120.6 (2)
C8—C7—C6	117.39 (15)	C16—C17—H17	119.7
C8—C7—H7A	108.0	C18—C17—H17	119.7
C6—C7—H7A	108.0	C17—C18—C13	120.41 (18)
C8—C7—H7B	108.0	C17—C18—H18	119.8
C6—C7—H7B	108.0	C13—C18—H18	119.8
H7A—C7—H7B	107.2	C36—C31—C32	118.80 (14)
C9—C8—C7	116.07 (15)	C36—C31—C3	121.03 (13)
C9—C8—H8A	108.3	C32—C31—C3	120.16 (13)
C7—C8—H8A	108.3	C33—C32—C31	120.83 (15)
C9—C8—H8B	108.3	C33—C32—H32	119.6
C7—C8—H8B	108.3	C31—C32—H32	119.6
H8A—C8—H8B	107.4	C34—C33—C32	118.37 (15)
C10—C9—C8	115.10 (16)	C34—C33—H33	120.8
C10—C9—H9A	108.5	C32—C33—H33	120.8
C8—C9—H9A	108.5	F1—C34—C33	118.67 (15)
C10—C9—H9B	108.5	F1—C34—C35	118.52 (16)
C8—C9—H9B	108.5	C33—C34—C35	122.81 (15)
H9A—C9—H9B	107.5	C34—C35—C36	118.38 (16)
C9—C10—C11	115.73 (14)	C34—C35—H35	120.8
C9—C10—H10A	108.3	C36—C35—H35	120.8
C11—C10—H10A	108.3	C31—C36—C35	120.80 (16)
C9—C10—H10B	108.3	C31—C36—H36	119.6
C11—C10—H10B	108.3	C35—C36—H36	119.6
H10A—C10—H10B	107.4	N2—C38—C2	175.68 (17)
C4—C11—C10	112.25 (13)	C1—N1—H1	109.5 (13)
C4—C11—H11A	109.2	C5—N3—C1	123.11 (12)
C10—C11—H11A	109.2	C5—N3—C12	120.92 (12)
C4—C11—H11B	109.2	C1—N3—C12	115.68 (12)
			
N1—C1—C2—C3	−179.54 (14)	C14—C15—C16—C17	1.1 (3)
N3—C1—C2—C3	0.16 (19)	C15—C16—C17—C18	−0.6 (3)
N1—C1—C2—C38	1.5 (2)	C16—C17—C18—C13	−0.6 (3)
N3—C1—C2—C38	−178.81 (12)	C14—C13—C18—C17	1.4 (3)
C38—C2—C3—C4	179.19 (13)	C12—C13—C18—C17	−174.81 (16)
C1—C2—C3—C4	0.3 (2)	C2—C3—C31—C36	−105.63 (18)
C38—C2—C3—C31	−0.3 (2)	C4—C3—C31—C36	74.87 (19)
C1—C2—C3—C31	−179.22 (13)	C2—C3—C31—C32	75.25 (18)
C2—C3—C4—C5	−0.2 (2)	C4—C3—C31—C32	−104.25 (17)
C31—C3—C4—C5	179.26 (13)	C36—C31—C32—C33	−0.4 (2)
C2—C3—C4—C11	−172.00 (13)	C3—C31—C32—C33	178.75 (14)
C31—C3—C4—C11	7.5 (2)	C31—C32—C33—C34	0.8 (2)
C3—C4—C5—N3	−0.3 (2)	C32—C33—C34—F1	179.75 (15)
C11—C4—C5—N3	171.40 (13)	C32—C33—C34—C35	−0.6 (3)
C3—C4—C5—C6	−179.76 (13)	F1—C34—C35—C36	179.53 (16)
C11—C4—C5—C6	−8.1 (2)	C33—C34—C35—C36	−0.2 (3)
C4—C5—C6—C7	87.41 (18)	C32—C31—C36—C35	−0.4 (3)
N3—C5—C6—C7	−92.12 (16)	C3—C31—C36—C35	−179.49 (16)
C5—C6—C7—C8	−73.7 (2)	C34—C35—C36—C31	0.6 (3)
C6—C7—C8—C9	67.0 (2)	C4—C5—N3—C1	0.7 (2)
C7—C8—C9—C10	−99.2 (2)	C6—C5—N3—C1	−179.73 (12)
C8—C9—C10—C11	54.5 (2)	C4—C5—N3—C12	−172.79 (13)
C5—C4—C11—C10	−89.02 (17)	C6—C5—N3—C12	6.73 (19)
C3—C4—C11—C10	82.57 (17)	N1—C1—N3—C5	179.05 (13)
C9—C10—C11—C4	53.53 (19)	C2—C1—N3—C5	−0.67 (18)
N3—C12—C13—C18	−47.2 (2)	N1—C1—N3—C12	−7.09 (19)
N3—C12—C13—C14	136.67 (15)	C2—C1—N3—C12	173.18 (11)
C18—C13—C14—C15	−0.9 (3)	C13—C12—N3—C5	−88.06 (16)
C12—C13—C14—C15	175.42 (16)	C13—C12—N3—C1	97.94 (15)
C13—C14—C15—C16	−0.3 (3)		

### Hydrogen-bond geometry (Å, º)

Cg1 is the centroid of the C13–C18 phenyl ring.

**Table:**

*D*—H···*A*	*D*—H	H···*A*	*D*···*A*	*D*—H···*A*
C32—H32···N1^i^	0.93	2.53	3.421 (2)	160
C11—H11*A*···*Cg*1^ii^	0.97	2.93	3.484 (2)	118

Symmetry codes: (i) −*x*+1, −*y*+1, −*z*+1; (ii) −*x*, −*y*+1, −*z*+1.
